# Systematic review and meta-analysis of dysregulated microRNAs derived from liquid biopsies as biomarkers for amyotrophic lateral sclerosis

**DOI:** 10.1016/j.ncrna.2024.02.006

**Published:** 2024-02-06

**Authors:** Hemerson Casado Gama, Mariana A. Amorós, Mykaella Andrade de Araújo, Congzhou M. Sha, Mirella P.S. Vieira, Rayssa G.D. Torres, Gabriela F. Souza, Janaína A. Junkes, Nikolay V. Dokholyan, Daniel Leite Góes Gitaí, Marcelo Duzzioni

**Affiliations:** a Laboratory of Pharmacological Innovation, Institute of Biological Sciences and Health, Federal University of Alagoas, Maceió, Alagoas -AL, 57072-900, Brazil; b Department of Cellular and Molecular Biology, Institute of Biological Sciences and Health, Federal University of Alagoas, Maceió, Alagoas -AL, 57072-900, Brazil; c Department of Biochemistry and Molecular Biology, Penn State College of Medicine, Hershey, PA, 17033, United States; d Postgraduate Program in Society, Technologies and Public Policies, Tiradentes University Centre, AL, 57038-000, Brazil; e Department of Pharmacology, Penn State College of Medicine, Hershey, PA, 17033, United States

**Keywords:** ALS, miRNAs, Biomarkers, Liquid biopsies, Dysregulated expression

## Abstract

The discovery of disease-specific biomarkers, such as microRNAs (miRNAs), holds the potential to transform the landscape of Amyotrophic Lateral Sclerosis (ALS) by facilitating timely diagnosis, monitoring treatment response, and accelerating drug discovery. Such advancement could ultimately improve the quality of life and survival rates for ALS patients. Despite more than a decade of research, no miRNA biomarker candidate has been translated into clinical practice. We conducted a systematic review and meta-analysis to quantitatively synthesize data from original studies that analyzed miRNA expression from liquid biopsies via PCR and compared them to healthy controls. Our analysis encompasses 807 miRNA observations from 31 studies, stratified according to their source tissue. We identified consistently dysregulated miRNAs in serum (hsa-miR-3665, -4530, -4745–5p, −206); blood (hsa-miR-338–3p, -183–5p); cerebrospinal fluid (hsa-miR-34a-3p); plasma (hsa-miR-206); and neural-enriched extracellular vesicles from plasma (hsa-miR-146a-5p, −151a-5p, −10b-5p, −29b-3p, and −4454). The meta-analyses provided further support for the upregulation of hsa-miR-206, hsa-miR-338–3p, hsa-miR-146a-5p and hsa-miR-151a-5p, and downregulation of hsa-miR-183–5p, hsa-miR-10b-5p, hsa-miR-29b-3p, and hsa-miR-4454 as consistent indicators of ALS across independent studies. Our findings provide valuable insights into the current understanding of miRNAs' dysregulated expression in ALS patients and on the researchers’ choices of methodology. This work contributes to the ongoing efforts towards discovering disease-specific biomarkers.

## Abbreviations

ALS: amyotrophic lateral sclerosis
ALS-FP: Fast-progressing ALS
ALSFRS/-R: ALS functional rating scale/-revised
ALS-PDC Kii Med Manual: Medical manual for ALS and Parkinsonism-dementia complex of the Kii Peninsula of Japan
ALS-SP: Slow-progressing ALS
AMOs: anti-miRNA oligonucleotides
CI: confidence interval
CSF: cerebrospinal fluid
ddPCR: digital drop PCR
ECM: Extracellular matrix
El-Escorial/-R: El-Escorial/-revised
ENMG: Electroneuromyography
EVs: extracellular vesicles
fALS: familial ALS
FDR: False Discovery Rate
FTD: Frontotemporal Dementia
HC: healthy control
K-ALS: Kii Peninsula ALS
KEEG: Kyoto Encyclopedia of Genes and Genomes
K-residents: Kii peninsula control residents
miRNAs: microRNAs
MND: motor neuron disease
N/I: Not informed
NEE: neural-enriched EVs
PCR: polymerase chain reaction
pre-miRNA: precursor miRNA
pri-miRNA: primary miRNA
RT-qPCR: quantitative reverse transcriptase PCR
sALS: sporadic ALS
SMIR: small molecular inhibitors
TLDA: TaqMan low-density array

## 1 Introduction

Amyotrophic lateral sclerosis (ALS) is a multisystemic and multifactorial disorder characterized by progressive degeneration of upper and lower motor neurons, leading to muscle atrophy, paralysis, and eventual death [1]. Key neuropathological features include extensive loss of lower motor neurons in the spinal cord and brainstem, degeneration of Betz cells in the primary motor cortex, deterioration of lateral corticospinal tracts, and reactive gliosis in degenerated areas of the motor cortex and spinal cord [2]. ALS manifests in two primary forms: familial ALS (fALS), defined by inherited mutations and accounting for around 10% of cases, and sporadic ALS (sALS), which has no known familial history, but can also harbor mutations found in fALS [3]. ALS's varied clinical outcomes from distinct mutations suggest it's a syndrome with multiple causes, while different phenotypes from one mutation imply a single mechanism influenced by complex gene-environment interactions [2]. Based on the somatic region involvement, patients can be further stratified into spinal-onset (70% of patients), which begins with limb muscle atrophy, or bulbar-onset (30% of patients), which starts with changes related to the cranial nerves that affect swallowing and vocal functions [1]. A recent meta-analysis reported a prevalence for all forms of ALS at 4.42 (95% CI 3.92–4.96) per million population and an incidence of 1.59 (95% CI 1.39–1.81) per million person-years [4].

The diagnosis and treatment of ALS pose significant challenges due to the absence of reliable biomarkers, the heterogeneous nature of the disease, and unclear underlying mechanisms. Currently, diagnosis relies on neurological assessment, detailed family history, and electromyography, coupled with the exclusion of mimicking diseases or alternative diagnoses [1,5]. However, this diagnostic process can be time-consuming, taking up to 16 months [6], and can result in misdiagnoses of patients who subsequently undergo unnecessary and invasive procedures, wasting valuable time for early disease management, drug treatment, and clinical trial enrollment [5,6]. Since median ALS survival time is approximately 3 years from symptom onset, this diagnostic delay could significantly increase disease burden and mortality [1]. The development of ALS biomarkers could enable timely and accurate diagnosis, aiding in tracking disease progression and therapeutic efficacy of drugs.

MicroRNAs (miRNAs) are a collection of small, conserved non-coding RNA molecules that hold great potential as biomarkers. These endogenous single-stranded fragments, ranging from 19 to 32 nucleotides in length [7], regulate gene expression by annealing to target mRNA sequences, thereby directing degradation or suppressing translation [8,9]. By negatively regulating the cell's transcriptome, miRNAs modulate cellular processes such as metabolism, proliferation, differentiation, survival, and apoptosis. Additionally, miRNAs can be loaded into extracellular vesicles (EVs) [10], acting as circulating signaling molecules, that influence not only the cell's activity and microenvironment but also distant tissues and organ homeostasis. Within the nervous system, miRNAs are involved in brain function, neurogenesis, and synaptic plasticity [11]. Their dysregulation or aberrant expression is linked to neurodegenerative diseases, including ALS [12,13]. Research indicates that the miRNAome is altered in various ALS tissues [14]. In particular, mutations in ALS-associated genes such as TARDBP and FUS are involved in miRNA biogenesis. TARDBP codes for the protein TDP-43, which binds directly to pri- and pre-miRNAs and interacts with the Drosha and Dicer complexes, which are significant interactions for neuronal outgrowth [15]. Additionally, TDP-43 cytoplasmic inclusions may sequester miRNAs, disrupting neuronal epigenetic homeostasis and contributing to motor neuron death [16]. FUS also possesses a dual function by interacting with pri-miRNA sequences and Drosha, and plays a relevant role in neuronal function, differentiation, and synaptogenesis [17]. Therefore, studying miRNAs dysregulation may provide novel insights into the pathomechanisms underlying ALS.

As biomarkers, miRNAs offer several advantages including disease and cell/tissue specificity, easy access through minimally invasive procedures, and molecular stability after prolonged storage, multiple freeze-thaw cycles, and in both fixed and paraffin-embedded samples. Moreover, they can be analyzed using accessible technology, such as PCR, microarrays, and RNA sequencing [18]. Despite these advantages and the urgent need for ALS biomarkers, no miRNA candidates have been successfully translated into clinical practice. Therefore, to contribute to the field, we conducted a systematic review to consolidate existing knowledge on dysregulated miRNA expression in liquid biopsies from ALS patients compared to healthy controls.

We report on 12 miRNAs with consistently dysregulated expression across different tissues, as identified through a systematic review. Subsequently, we synthesized the evidence for the dysregulation of each miRNA by a meta-analysis, with the primary endpoint of relative fold-change in ALS patients compared with healthy controls. Using meta-analytic techniques, we provide conservative estimates and 95% confidence intervals for the relative fold-changes for each of the dysregulated miRNAs. Pathway enrichment analysis revealed an overrepresentation of signaling pathways linked to ALS pathomechanisms. Our findings shed light on the efforts, study designs, and results aimed at discovering miRNAs as biomarkers for ALS, and present a comprehensive list of all miRNAs screened to date, offering valuable insights for future methodological designs. Moreover, we emphasize the relevance of disease phenotypic stratification, accurate clinical diagnosis, methodological standardization, and the deposit of miRNA findings using standardized methodologies into international databases to facilitate data sharing.

## 2 Methods

The protocol for this study adhered to the Preferred Reporting Items for Systematic Reviews and Meta-analysis (PRISMA) guidelines [19], and its details are registered in the International Prospective Register of Systematic Reviews (CRD42021230232)

### 2.1 Literature search

We performed a comprehensive query search of PubMed, Embase, Web of Science, and Virtual Health Library databases, without language restrictions (Table 1). To ensure the thoroughness of our search, we manually screened the references from the studies we selected for qualitative analysis and recent reviews on the topic. Additionally, we employed ResearchRabbit (https://researchrabbitapp.com), a literature mapping tool that facilitates the discovery of related publications (Fig. S1).

**Table 1:** Search strategies employed for PubMed, Embase, Scopus, and Virtual Health Library databases (Jan. 2000 to Dec. 2022).

Database	Research query	N of articles retrieved
PubMed	(((miRNA) OR (microRNA)) AND ((“Motor Neuron Disease”) OR (“Amyotrophic Lateral Sclerosis”))) AND ((Human) OR (Patient))	326
Embase	(miRNA OR microRNA) AND (“motor neuron disease” OR “amyotrophic lateral sclerosis”) AND (human OR patient)	961
Scopus	((TITLE-ABS-KEY (mirna) OR TITLE-ABS-KEY (microrna))) AND ((TITLE-ABS-KEY (“motor neuron disease”) OR TITLE-ABS-KEY (“amyotrophic lateral sclerosis”))) AND ((TITLE-ABS-KEY (human) OR TITLE-ABS-KEY (patient)))	815
Virtual Health Library	(miRNA) OR (microRNA) AND (“Motor Neuron Disease”) OR (“Amyotrophic Lateral Sclerosis”) AND (human) OR (patient)	310

### 2.2 Study selection

We used Rayyan (https://rayyan.qcri.org) [20] for the systematization of the screening process. After removing duplicates (HCG, MPSV, RGDT, JAJ, and GFS), four independent reviewers (HCG, MPSV, JAJ, and RGDT) assessed the titles and abstracts. Disagreements were resolved by two additional reviewers (MAA and DLGG). We applied four exclusion criteria: 1) non-original studies; 2) non-ALS studies; 3) non-human studies; and 4) non-miRNA expression studies. Articles that passed this initial screening were fully read and subject to our eligibility criteria: 1) patient studies (i.e.: non *in vitro* studies); 2) biological fluids as source material; 3) ALS *versus* healthy controls; 4) PCR evaluation or validation of miRNA expression; and 5) clear statistical analysis.

### 2.3 Data extraction

Two independent reviewers (MAA and MAdA) extracted data from eligible studies in the chronological order of publication. Disagreements were discussed, and if consensus could not be reached, two additional reviewers assisted (HCG and DLGG). Information was organized into an Excel spreadsheet (Microsoft Corporation), which included the following: 1) article identification; 2) clinical data of ALS patients; 3) information regarding healthy controls; and 4) sample collection data [[21], [22], [23], [24], [25], [26], [27], [28], [29], [30], [31], [32], [33], [34], [35], [36], [37], [38], [39], [40], [41], [42], [43], [44], [45], [46], [47], [48], [49], [50], [51]] (Table S1). We created a second document containing the following information: 1) miRNA name; 2) its expression compared to control (upregulated, downregulated, or unaltered); 3) the number of ALS patients and healthy controls; 4) the tissue source; and 5) the article reference (Table S2). We reached out to authors if a miRNA was studied more than once but did not specify whether it belonged to the 3′ or 5’ hairpin arms. The fold change in miRNA expression was extracted from the publications using either the numbers as reported in the manuscript text or tables, or from manuscript figures using a data extraction tool (https://automeris.io/WebPlotDigitizer).

We excluded 12 miRNA observations (hsa-miR-133a-3p, hsa-miR-135b-5p, hsa-miR-143–3p, hsa-miR-144–3p, hsa-miR-146b-3p, hsa-miR-206, hsa-miR-20a-3p, hsa-miR-214–3p, hsa-miR-331–3p, hsa-miR-374b-5p, hsa-miR-518d-3p, and hsa-miR-551b-3p) from a discovery cohort consisting of 24 sALS patients and 25 healthy controls [32] due to our uncertainty regarding the direction of their dysregulation. Furthermore, we excluded 21 miRNA observations (hsa-let-7b-5p, hsa-let-7d-3p, hsa-let-7d-5p, hsa-miR-126–3p, hsa-miR-126–5p, hsa-miR-133a-3p, hsa-miR-1-3p, hsa-miR-143–3p, hsa-miR-146a-3p, hsa-miR-194–3p, hsa-miR-23a-3p, hsa-miR-330–3p, hsa-miR-338–3p, hsa-miR-339–3p, hsa-miR-339–5p, hsa-miR-451a, hsa-miR-517a-3p, hsa-miR-584–5p, hsa-miR-625–3p, hsa-miR-708–5p, and hsa-miR-744–5p) screened in neural-enriched EVs (NEE) from plasma from 10 ALS patients and 10 healthy controls. This data was presented as either non-significantly expressed between patients and healthy controls or showing significant differences in only one of the two experiments conducted with different patient cohorts [40]. Thus, we were unable to identify the direction of dysregulation as individual datasets.

### 2.4 Meta-analysis

We (CMS and MAA) started with the 12 miRNAs found in our systematic review to be consistently dysregulated. We excluded miRNAs for which fewer than three comparisons were reported in the literature. Not all studies reported miRNA expression levels separately for ALS vs control samples; therefore, we used only the ratio of miRNA expression level (ALS divided by control) in our meta-analyses; we refer to this ratio as the fold change.

Since fold changes have a hard cutoff on the left at 0 and are asymmetric depending on which group is used as reference, potentially leading to significant skew of the underlying distributions, we used the log_2_ of the fold change as our primary endpoint. We assumed that the log_2_-fold changes were normally-distributed. Theoretically, this approach is further justified because the ratio of two normally distributed variables (e.g.: ALS miRNA expression and healthy control miRNA expression) is a Cauchy random variable, whose variance is infinite or undefined. In contrast, assuming the logarithms are normally-distributed, then the log of the ratio is a difference of Gaussian variables (log_2_ A/B = log_2_ A – log_2_ B) which possesses a well-defined variance.

Since there was heterogeneity among studies in the reporting of variances on the estimated fold change (some reported variances for ALS and control separately, others did not report a variance), we used a model which estimates study variance using solely the study sample size [52], which we defined as the total number of patients (ALS and control).

For our meta-analyses, we included only those studies that reported a statistically significant dysregulation of the miRNA under consideration. If we allowed all studies to be included, with non-significant results reported as no fold change (fold change equal to 1), the models do not converge. The statistical question we answered was therefore: “Given a population of studies in which the miRNA was found to be significantly dysregulated, what is the maximum likelihood estimate of the log_2_-fold change for that miRNA in ALS patients vs healthy controls.”

To estimate the probability that a dysregulated miRNA was reported as a false positive, we adopted the following model: For each miRNA *m*, we define the indicator variable *S*_*m*_, such that *S*_*m*_ is 1 if *m* was found to be statistically significantly dysregulated and 0 otherwise. We assume that the result of each study for the *m* is drawn from the distribution of *S*_*m*_. Then we can analyze the resulting contingency table using Boschloo's exact test [53], to test if the true proportion of statistically significant studies is different from 0:

$$\left[\begin{array}{cc} \#\;\text{of}\;\text{significant}\;\text{studies} & \#\;\text{total}\;\text{studies}\;‐\;\#\;\text{of}\;\text{significant}\;\text{studies}\\ 0 & \#\;\text{total}\;\text{studies} \end{array}\right]$$

We assessed for publication bias using the p-curve method [54], and we assessed for heterogeneity using Cochran's Q test [55], with p < 0.10 indicating the presence of significant heterogeneity, and with the I^2^ statistic [56], with I^2^ > 0.25 indicating significant heterogeneity.

We used Python 3.11 to perform meta-analysis, with the PyMARE 0.0.4 [57] as the regression engine and computation of Cochran's Q test and the I^2^ statistic, pypcurve 0.1.0 [54] for the p-curve method, Pandas 2.0.1 [58] to organize our data, and the myforestplot package (https://github.com/toshiakiasakura/myforestplot) for plotting. Jupyter Notebooks containing the code necessary to reproduce our analysis and forest plots are available as Supplemental Material (**Code and Data availability**).

### 2.5 Bioinformatics analysis

We (MAdA and HCG) performed pathway enrichment analysis of the 12 consistently dysregulated miRNAs using DIANA miR-Path v.3 (http://www.microrna.gr/miRPathv3) to identify enriched pathways targeted by miRNAs [59]. Experimentally validated human target genes from the Tarbase v7.0 database were included, and Fisher's exact test with a Pathway union category, false discovery rate (FDR) correction, and a p-value threshold of ≤0.05 was used. TargetScan context score at −0.4 was also utilized. Functional annotation was performed using the Kyoto Encyclopedia of Genes and Genomes (KEGG) databases.

## 3 Results

Our search retrieved 2412 studies, of which 1344 duplicates were eliminated. We applied our exclusion criteria to the remaining 1068 studies, resulting in the additional removal of 971 studies. After full-text screening of the remaining 97 studies, we identified 31 as eligible for qualitative synthesis. A manual search did not yield additional records (Fig. 1A).

**Fig. 1:**
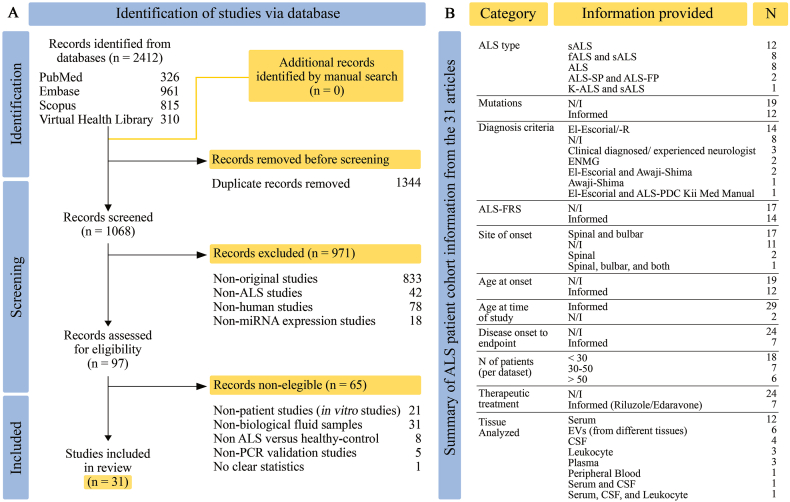
Data workflow and characteristics of ALS patients. **(A)** Flow diagram following the PRISMA guideline (2020) for the article selection process. The last manual search was conducted on January 30, 2023, which did not yield new records. **(B)** Summary from Table S1 on the extracted data from the 31 selected articles. Abbreviations: N/I, not informed; ALS-FP, fast-progressing ALS; ALS-SP, slow-progressing ALS; ALSFRS/-R, ALS functional rating scale/-revised; K-ALS, Kii peninsula ALS; EVs, extracellular vesicles; CSF, cerebrospinal fluid; ALS-PDC Kii Med Manual, medical manual for ALS and Parkinsonism-dementia complex of the Kii Peninsula of Japan; ENMG, Electroneuromyography; El-Escorial/-R, -revised.

### 3.1 Variations between studies on population and samplings

The studies included in this systematic review examined the differential expression of miRNAs in biological fluids from ALS patients compared with healthy controls. Most studies focused on sALS (38.7%) [21,22,24,26,[29], [30], [31], [32], [33],[36], [37], [38]], and few reported disease-associated mutations (38.7%) [25,27,31,33,35,37,41,42,46,47,49,51]. Most studies used El-Escorial, or its revised version, as diagnostic criteria (45.2%) [22,23,[25], [26], [27],[29], [30], [31],^33^,^35^,^40^,^42^,^48^,^50^], while others relied on a ‘clinical’ or ‘experienced neurologist’ assessment (9.7%) [41,47,51] without further details, or did not provide any information (25.8%) [32,34,36,37,39,[44], [45], [46]]. Most cohorts consisted of spinal- and bulbar-onset patients (54.8%) [[24], [25], [26], [27],[29], [30], [31], [32], [33],[37], [38], [39],^42^,^43^,^47^,^48^,^51^], followed by studies which did not provide information (35.5%) [[21], [22], [23],[34], [35], [36],^40^,[44], [45], [46],^50^]. Among spinal-onset patients, some studies differentiated between upper- and lower-limb manifestations [26,30,32,38]. Furthermore, fewer than half of the studies reported on the age of disease onset (38.7%) [24,25,27,28,[30], [31], [32], [33],^37^,^42^,^43^,^48^,^49^], and the ALS functional rating scale, or its revised version (ALSFRS/-R; 45.2%) [22,[25], [26], [27], [28], [29], [30],^33^,[38], [39], [40], [41],^44^,^48^]. Only four studies did not report on the age of the patients at the time of study (12.9%) [34,37,40,50]. Regarding sample size, most studies included fewer than 30 patients (58.1%) [[21], [22], [23],^25^,^27^,^29^,^31^,^32^,^34^,^35^,[38], [39], [40], [41],[43], [44], [45], [46], [47]], with one study comprising of only seven individuals [47]. The remaining studies consisted of 30–50 patients (22.6%) [26,28,33,36,[48], [49], [50], [51]], and more than 50 patients (19.4%) [24,30,37,42,49] with the largest cohort consisting of 84 patients [37]. Information on therapeutical treatment was largely absent, with only seven studies providing any form of information (22.6%) [31,32,35,37,38,40,47]. The primary source of tissue sampling was serum (38.7%) [23,25,27,29,32,35,38,[41], [42], [43],^45^,^46^], followed by EVs derived from different fluids (19.4%) [34,39,40,44,47,50], CSF (12.9%) [31,36,49,51], leukocytes (9.7%) [21,30,37], plasma (9.7%) [26,28,48], and peripheral blood (3.2%) [33]. Only two studies analyzed multiple tissues, including serum and CSF (3.2%) [22]; and serum, CSF, and leukocytes (3.2%) [24] (Fig. 1B, Table S1).

Information on other sample characteristics was limited, with only a few studies informing the time elapsed from disease onset to sampling (35.5%) [24,26,30,31,33,38,39,41,47,49,51], or the time at collection (16.1%) [21,24,30,32,49]. All studies reported on the number of healthy controls, and most on sex (83.9%) [[21], [22], [23], [24], [25], [26], [27], [28],[30], [31], [32], [33],[35], [36], [37], [38], [39],^41^,[43], [44], [45], [46], [47], [48],^50^,^51^] and age (90.3%) [[21], [22], [23], [24], [25], [26], [27], [28], [29], [30], [31], [32], [33],[35], [36], [37], [38], [39],^41^,[43], [44], [45], [46], [47], [48], [49], [50], [51]]. Additionally, age- and sex-matching to ALS patients was mentioned in 38.7% of the studies [21,24,25,27,28,30,31,38,41,42,46,48] (Table S1).

### 3.2 Differential deregulatory expression of miRNAs between studies and meta-analysis

We analyzed data on 807 miRNA species from 31 studies (Table S2) and stratified them based on tissue source and direction of dysregulation (i.e.: up-, down-, or unregulated) (Table S3). We defined a miRNA as inconsistent when its expression showed contradictions between independent datasets. Furthermore, while we report on miRNAs represented by a single dataset, we do not include them in our analysis to ensure higher reliability and robustness of our findings.

The most frequently assessed tissue was serum, with 789 miRNAs analyzed. Among these, 522 were screened only once, and 77 showed inconsistent results. We identified 186 consistently unregulated miRNAs, three consistently downregulated (hsa-miR-3665, hsa-miR-4530, and hsa-miR-4745–5p), and one consistently upregulated (hsa-miR-206). Peripheral blood and leukocytes were regarded as one tissue, presenting 60 miRNAs, of which 57 were analyzed only once, one showed inconsistent result, one consistently downregulated (hsa-miR-183–5p) and one consistently upregulated (hsa-miR-338–3p). CSF was assessed for 43 miRNAs, with 29 presenting one dataset, five showing inconsistency, eight consistently unregulated, and one consistently upregulated (hsa-miR-34a-3p). Plasma was examined for 13 miRNAs, from which tissue, 11 were analyzed only once, one was inconsistent and one was consistently upregulated (hsa-miR-206). EVs originating from the aforementioned tissues were considered as separate datasets. Serum exosomes were analyzed for 11 miRNAs, CSF exosomes for two, and plasma EVs for seven. All of these miRNAs were analyzed only once. The data from plasma NEE presented more robust evidence with eight miRNAs screened. Among these, three showed inconsistent dysregulations, while the remaining five were consistent, two upregulated (hsa-miR-146a-5p and hsa-miR-151a-5p), and three downregulated (hsa-miR-10b-5p, hsa-miR-29b-3p, and hsa-miR-4454) (Fig. 2A, Table 2, Table S3).

**Fig. 2:**
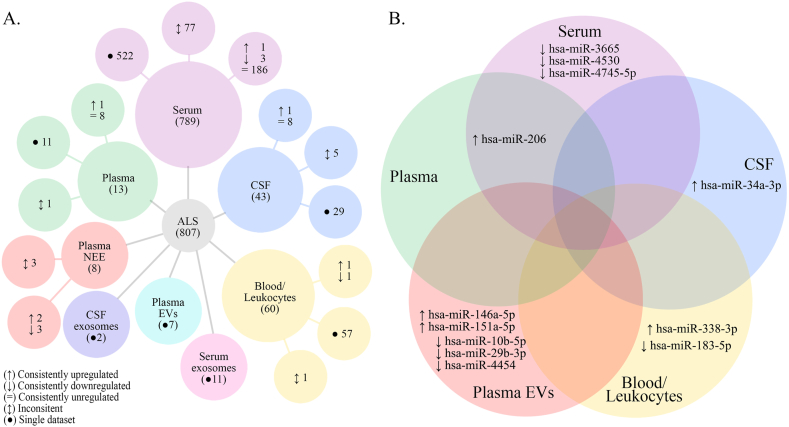
Synthesis of screened miRNAs from different tissues. **(A)** Representation of miRNAs screened in ALS liquid biopsies. **(B)** Overlap of consistently dysregulated miRNAs, either up- or downregulated, across different tissues. The analysis includes miRNAs with at least two independent datasets. Abbreviations: EVs, extracellular vesicles; CSF, cerebrospinal fluid; NEE, neural-enriched EVs.

**Table 2:** Summary from Table S3 on the consistently reported up- and down-regulated miRNAs stratified by tissue. Abbreviations: CSF, cerebrospinal fluid; HC, healthy control; NEE, neural-enriched EVs. Symbols: ↑, upregulated; ↓, downregulated; #, Authors kindly provided information on the miRNA’ -3p or -5p.

Tissue	Axis	microRNA	Type ALS	ALS (N)	HC (N)	Author (year)	Reference
Serum	↑	hsa−miR−206	sALS	23	22	Waller (2017)	[32]
				14	8	Tasca (2016)	[29]
			ALS	12	12	Toivonen (2014)	[23]
				14	17	Malacarne (2021)	[46]
	↓	hsa−miR−3665	fALS	13	13	Freischmidt (2014)	[25]
			sALS	14	14		
		hsa−miR−4745−5p	fALS	13	13		
			sALS	14	14		
		hsa−miR−4530	fALS	13	13		
			sALS	14	14		
Peripheral Blood/Leukocytes	↓	hsa−miR−183−5p	sALS	83	61	Chen (2016) #	[30]
				50	15	Liguori (2018)	[33]
	↑	hsa−miR−338−3p		14	14	De Felice (2012)	[21]
				72	62	De Felice (2014)	[24,37]
				20	20		
				84	27	Vrabec (2018)	[37]
CSF	↑	hsa−miR−34a−3p	ALS	55	19	Rizzuti (2022)	[49]
			fALS	27	19		
Plasma	↑	hsa−miR−206	ALS	39	39	Andrade (2016)	[28]
				30	20	Soliman (2021)	[48]
Plasma NEE	↑	hsa−miR−146a−5p	ALS	10	10	Banack (2020)	[40]
				10	10		
				50	50	Banack (2022)	[50]
		hsa−miR−151a−5p		10	10	Banack (2020)	[40]
				10	10		
				50	50	Banack (2022)	[50]
	↓	hsa−miR−10b−5p		10	10	Banack (2020)	[40]
				10	10		
				50	50	Banack (2022)	[50]
		hsa−miR−29b−3p		10	10	Banack (2020)	[40]
				10	10		
				50	50	Banack (2022)	[50]
		hsa−miR−4454		10	10	Banack (2020)	[40]
				10	10		
				50	50	Banack (2022)	[50]

To identify dysregulated miRNAs shared across different tissues, we focused only on those consistently reported as up- and downregulated. We found that hsa-miR-206 upregulation was detected in both serum and plasma. Although also reported in leukocytes, we excluded this dataset due to its limited representation (Fig. 2B).

To provide rigorous and standardized quantification of the evidence for eight of the 12 miRNAs, for which at least three values were reported for each, we performed meta-analysis on the log_2_ fold changes of each miRNA in ALS samples vs healthy control.

**Fig. 3:**
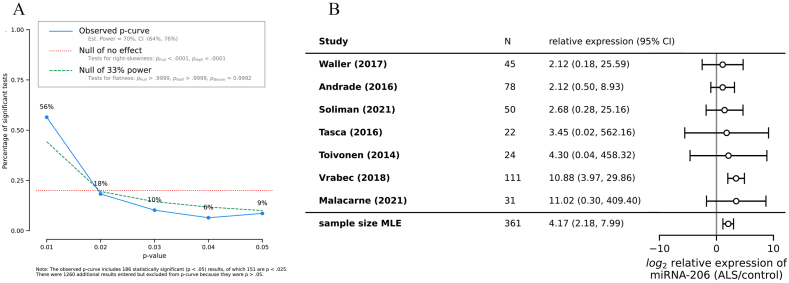
Meta-analysis publication bias and forest plot. **(A)** The result of p-curve analysis on statistically significant miRNA results reported from all 31 studies, demonstrating low publication bias and high study power. **(B)** The forest plot of the estimated log_2_ fold change for hsa-miR-206 in ALS samples vs healthy control, demonstrating a consistent upregulation despite conservatively high estimates of within-study variance. The last line of the forest plot is our estimated effect size.

We assessed for publication bias using the p-curve method (Fig. 3A). P-curve analysis posits that false positives will be reported with p-values that are uniformly distributed in the range p < 0.05, whereas a true effect tends to be reported with low p-value (p < 0.01) more often than with values close to p = 0.05. Here, we pooled all the p-values reported across 31 studies, and conclude that there is a low likelihood of publication bias or p-manipulation, since the observed p-curve demonstrates an estimated power of 70% (95% CI: 64%–76%), and we reject the null hypothesis of there being no true effects with p < 0.001.

We performed a sample size-based estimation of within-study variance and fit a random effects model to our data for each miRNA with greater than two observations within the same tissue (Methods). We summarize all the miRNAs that were analyzed (Table 3), and the remaining forest plots are included in Fig. S2.

**Table 3:** Results of meta-analysis on estimated fold change in expression in ALS vs healthy control, for miRNAs with >2 values reported. *95% CI does not overlap with 1. #Values were published by a single research group across multiple publications. In all the analyses, the I^2^ statistic was 0%.

miRNA	Estimated fold change in ALS vs control	95% CI	Number of observations	Cochran's Q test (p-value)	False positive rate
hsa-miR-206*	4.17	(2.18, 7.99)	7	4.94 (0.42)	0.007
hsa-miR-206 (serum-only)	4.44	(1.80, 10.95)	4	1.95 (0.37)	0.066
hsa-miR-338–3p	2.46	(1.06, 5.71)	3	0.64 (0.42)	0.341
hsa-miR-183–5p*	0.30	(0.14, 0.64)	3	0.22 (0.63)	0.341
hsa-miR-146a-5p*#	1.29	(1.14, 1.45)	3	0.58 (0.44)	0.341
hsa-miR-151a-5p#	2.12	(0.90, 5.01)	3	0.62 (0.42)	0.341
hsa-miR-10b-5p#	0.31	(0.09, 1.05)	3	0.57 (0.45)	0.341
hsa-miR-29b-3p*#	0.59	(0.39, 0.89)	3	0.37 (0.54)	0.341
hsa-miR-4454*#	0.49	(0.30, 0.77)	3	0.34 (0.55)	0.341

When we defined consistent regulation of a miRNA as more than two published manuscripts from independent groups reporting the same direction, only hsa-miR-206 met this criterion (Fig. 3B). From our meta-analysis, we found evidence for consistent upregulation of hsa-miR-206, hsa-miR-146a-5p, hsa-miR-151a-5p, and hsa-miR-338–3p, and consistent downregulation of hsa-miR-183–5p, hsa-miR-10b-5p, hsa-miR-29b-3p, and hsa-miR-4454. As a caveat, many of the miRNAs (−146a-5p, −151a-5p, −10b-5p, −29b-3p, and −4454) were reported in more than two observations by a single research group across multiple publications [40,50], and were therefore not consider independently validated (Table 3). The remaining four miRNAs (−34a-3p, −3665, −4530, and -4745–5p) were represented by only two datasets from the same authors and same manuscript and were thus excluded from this analysis.

Three of our statistically significant analyses (hsa-miR-206, hsa-miR-183–5p, hsa-miR-338–3p) pooled data from independent research groups. Notably, the analysis for hsa-miR-206 mantained its statistical significance after we restricted the inclusion to serum only-studies (Fig. S2A). We did not find evidence of significant publication heterogeneity by either Cochran's Q test or the I^2^ statistic, with the caveat that few studies were analyzed. We estimated the false positive rate for each miRNA using Boschloo's exact test. In each contingency table, we used the number of significant studies, rather than the number of significant values reported by studies.

### 3.3 Enriched pathways targeted by consistently reported miRNAs

Since each miRNA can regulate multiple transcript genes and each target gene can be regulated by several miRNAs, biological regulatory pathways may play a crucial role in the pathomechanisms of ALS. To investigate potential pathways, we explored the connection between functional categories and analysis of enriched pathways associated with consistently dysregulated miRNAs.

**Fig. 4:**
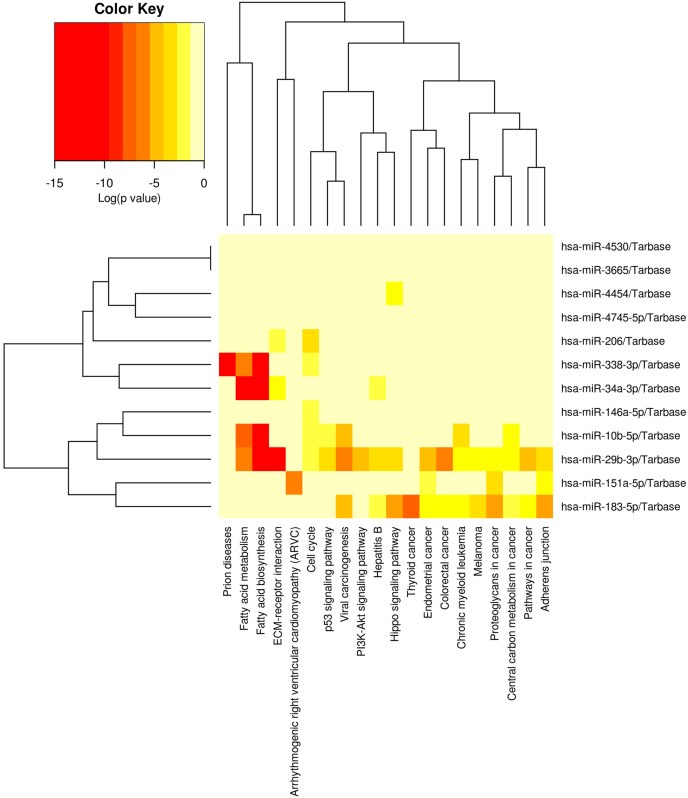
The heatmap illustrates the KEGG pathways associated with the 12 consistently identified miRNAs. The gradient color scheme indicates the strength of association, with closer interactions shown in red (lower p-value).

Our analysis showed a significant association with several processes and diseases. Some pathways showed strong association, p-value 0.001 or lower, with prion disease, extracellular matrix (ECM)-receptor interaction, fatty acid biosynthesis and metabolism. Other pathways such as adherens junction, cell cycle, viral carcinogenesis, Hippo signalling pathway, proteoglycans in cancer, colorectal cancer, endometrial cancer, thyroid cancer, chronic myeloid leukemia, hepatitis B, p53 signaling pathway, pathways in cancer, central carbon metabolism in cancer, arrhythmogenic right ventricular cardiomyopathy, PI3-Akt signaling pathway, and melanoma also showed significant moderate associations, as indicated by p-values <0.05 (Fig. 4).

## 4 Discussion

Despite extensive research into the dysregulated expression of miRNAs, identifying a disease-specific miRNAs or a diagnostic panel for ALS remains elusive. To address this gap, we conducted a systematic literature review and reported on the differential miRNA expression in biological fluids between ALS patients and healthy controls via RT-qPCR. Under these premises, only 1.28% of all studies met our eligibility criteria, and of these, merely 1.48% of the assessed miRNAs showed consistent dysregulation. This low percentage can be attributed to several factors, including the representation of many miRNAs by individual datasets and the inconsistencies in expression across studies, potentially stemming from methodological variations.

The broad spectrum of pathological processes with complex genotypic and phenotypic variables encompassed by ALS [60] contributes to the variability observed in miRNA expression. By grouping these variables, the identification of a specific molecular signature can be obscure, and thus, careful patient stratification is crucial.

### 4.1 Epidemiological variations

#### **4.1.1** Diagnosis criteria and ALS subtype (fALS vs sALS)

Our analysis revealed differences in the diagnostic approaches across studies, with less than half adhering to El-Escorial criteria [61] or its revised version [62]. Unexpectedly, some studies did not report any criteria, prompting us to include data from any population referred to as ‘ALS’ by the authors. Most studies focused on sALS patients, with limited comparisons to fALS. Freischmidt et al. analyzed serum samples from fALS (n = 13) and sALS (n = 14) patients, each matched to healthy controls, reporting a highly similar miRNA expression pattern. They found shared downregulation of hsa-miR-4745–5p, hsa-miR-3665, and hsa-miR-4530, with exclusive downregulation of hsa-miR-1915–3p in fALS [63]. In a subsequent study, sALS (n = 20) presented downregulation of hsa-miR-1234–3p, and shared downregulation of hsa-miR-1825 with fALS (n = 13) [27]. Raheja et al. reported higher expression of hsa-miR-574–3p in sALS (n = 20), and a lower expression of hsa-miR-628–3p in fALS (n = 3) [35]. Dobrowolny et al. found downregulation of hsa-miR-151a-5p, hsa-miR-199a-5p, and hsa-miR-423–3p in both fALS (n = 4) and sALS (n = 19) versus healthy controls (n = 11) [45]. In plasma, Soliman et al. observed increased levels of hsa-miR-206, hsa-miR-142–3p, hsa-miR-143–3p and hsa-miR-106, and decreased levels of hsa-miR-4516 and hsa-let-7f-5p in fALS (n = 8) compared to sALS (n = 22) [48]. In CSF, Rizzuti et al. showed higher expression of hsa-miR-625–3p in fALS (n = 27) versus sALS (n = 28) [49]. The existing evidence reveals a significant gap in understanding miRNA differences between sALS and fALS, limited by few studies, small patient population, and lack of replicated findings for a specific miRNA from a specific tissue. Future research should explore these variances prioritizing diagnostic criteria standardization to enhance the homogeneity within study groups and reduce the influence of confounding factors.

#### 4.1.2 Genotyping stratification

Frequently, studies failed to report or stratify patient cohorts based on mutations, potentially due to the resource-intensive nature of genetically screening each sample or the limited availability of recruitable patients with shared specific mutations. For instance, Benigni et al., dichotomized ALS into C9orf72 expansion carriers (n = 8) and non-carriers (n = 16), analyzing a pattern of eight miRNAs from CSF (hsa-let-7a-5p, hsa-let-7b-5p, hsa-let-7f-5p, hsa-miR-15b-5p, hsa-miR-21–5p, hsa-miR-148a-3p, hsa-miR-181a-5p, and hsa-miR-195–5p). They found no significant differences, nor when comparing groups based on sex, onset, or age at onset [31].. Notably, Freischmidt et al. screened a group of asymptomatic mutation carriers (n = 18) and compared them to healthy controls (n = 8), revealing a 91.7% overlap in miRNA profile with fALS (n = 9) compared to healthy controls (n = 10) [63]. Genetic stratification holds great potential for uncovering differential miRNA patterns. For example, TARDBP and FUS mutations code for RNA-binding proteins involved in miRNA processing [[15], [64]]. Creating an efficient genotyping workflow as a standard procedure can be difficult for laboratories centered on posttranslational studies. Nonetheless, collaborating with hospitals and ALS networks can help to consolidate resources and facilitate recruitment of patients with similar mutations. This approach enables the exploration of common genetic pathways between fALS and sALS beyond traditional inheritance patterns. Integrating genotyping data with miRNA profiling can provide valuable new insights into the role of miRNAs in ALS pathogenesis.

#### 4.1.3 Exclusion criteria and comorbidities

While several studies implemented exclusion criteria or provided information on comorbidities [26,28,33,38,40,48], only one study included patients with Alzheimer's symptoms (n = 2) and frontotemporal dementia (FTD; n = 4) [37]. Another study classified their cohort as ALS-FTD [42]. Given the potential symptom overlap between ALS and other neurological diseases, particularly in early stages, and the existence of shared mutations between FTD and ALS [65], it is crucial for researchers to adopt standardized diagnostic criteria and diligently report any comorbidities. Considering these factors, studying well defined cohorts comprising ALS patients, and those with comorbidities, can serve as a strategy to identify biomarkers capable of distinguishing between different presentations of ALS.

#### 4.1.4 Site of onset (spinal-onset vs bulbar-onset)

The site of onset significantly affects the disease's clinical course and influences therapeutic intervention choices. Patients with bulbar-onset, in particular, have a worse prognosis and shorter survival rate due to early respiratory dysfunctions [66], making it highly relevant to explore their miRNA signature. Takahashi et al. found decreased levels of hsa-let-7f-5p in the plasma of spinal-onset patients (n = 27) compared with bulbar-onset (n = 20) [26]. Tasca et al. reported higher expression of hsa-miR-133a, hsa-miR133b, and hsa-miR-206 in spinal-onset (n = 10), whereas bulbar-onset (n = 3) presented higher levels of hsa-miR-155; both groups shared similar levels of hsa-miR-1, hsa-miR-146a, hsa-miR-149, hsa-miR-221, hsa-miR-27a [29]. In serum, Waller et al. found no differences in hsa-miR-206, hsa-miR-143–3p, or hsa-miR-374b-5p between bulbar-onset (n = 9), upper-limb-onset (n = 7), and lower-limb-onset (n = 7) [32]. In peripheral blood, Ligouri et al. were able to distinguish spinal-onset (n = 36) patients from bulbar-onset (n = 14) cases based on the downregulated expression of hsa-miR-106b-3p, hsa-miR-128–3p, hsa-miR-148b-3p, hsa-miR-186–5p, hsa-miR-30b-5p, hsa-miR-30c-5p, and hsa-miR-342–3p [33]. Lastly, in plasma, Soliman et al., found that spinal-onset (n = 21) had lower levels of hsa-miR-142–3p and hsa-miR-143–3p, but higher levels of hsa-miR-106 and hsa-miR-4516, compared to those with bulbar-onset (n = 9), with no differences regarding hsa-let-7f-5p and hsa-miR-206 [48]. Dispite clinical importance, we found that studies comparing miRNA expression across these disease manifestations are scarce.

#### 4.1.5 Differences in sex, disease duration, and age at onset

We found scarce data on miRNAs differential expression across sex and disease duration, with an absence of information regarding age at onset. Toivonen et al. observed a distinct miRNA expression pattern in serum from female ALS patients. Initially, they evaluated ALS patients (n = 12) vs healthy controls (n = 12), reporting upregulation of hsa-miR-106b and hsa-miR-206 in the ALS group. When they divided the group based on sex, ALS females (n = 6) against their matched healthy controls, they showed significantly increased levels of hsa-miR-145, hsa-miR-133b, and hsa-miR-206. While the male group only showed significant dysregulation of high levels of hsa-miR-206 [23]. In contrast, Yelick et al. found no differences between sex in the expression of hsa-miR-124–3p from CSF exosomes or when comparing age and weight/height [44]. Given hormonal differences, and their association with ALS [67], it becomes an interesting venue to further explore. As for disease duration, De Felice et al. reported a significant positive correlation with the expression levels of miR-338–3p in leukocytes [24]. In serum, Raheja et al. showed lower expression (associated with longer disease duration) of hsa-miR-142–3p, hsa-miR-21–5p, hsa-miR-33a-5p, hsa-miR-34a-5p, hsa-miR-376b-3p, and hsa-miR-491–5p, and elevated expression of hsa-miR-9-3p [35]. It is likely that different miRNAs emerge or show varying degrees of dysregulation at distinct disease stages, underscoring the importance of reporting the timing and age of symptoms onset. Furthermore, considering the existence of a juvenile form of the disease, we advocate for age group patient stratification.

#### 4.1.6 Disease progression and follow-ups

To track and measure disease progression and predict survival time, researchers commonly use the ALSFRS/-R [68]. This scale serves as a valuable tool allowing to correlate molecular cues to therapeutic interventions and enables comparisons between patients in the same disease stages. Initial attempts to correlate miRNA dysregulation with ALSFRS were conducted by Freischmidt et al., in CSF and serum, but yielded no significant results [22]. In plasma, Takahashi et al. found a negative correlation between hsa-let-7f-5p and the ALSFRS-R bulbar paralysis score [26]. In the same tissue, de Andrade et al. reported no differences in hsa-miR-206 and hsa-miR-424 between mild/early (>24) to severe/late (<25) stages [28]. In serum, Matamala et al. observed a negative correlation between increasing ALSFRS scores and the expression levels of hsa-miR-142–3p, but not hsa-miR-1249–3p [38]. In the same tissue, Reheja et al. identified a significant correlation between ALSFRS-R scores and high expression of hsa-miR-2110 (associated with a more rapid disease progression), as well as low expression of hsa-miR-136–3p, hsa-miR-30b-5p, hsa-miR-331–3p, and hsa-miR-496 [35]. From serum exosomes, Saucier et al., could distinguish between low (<30) and high (≥30) scores analyzing hsa-miR-193a-5p expression [39]. In CSF exosomes, Yelick et al. reported upregulation of hsa-miR-124–3p correlating with lower ALSFRS-R scores in male patients (n = 9) versus females (n = 5), while there was no correlation with hsa-let-7c [44].

Two studies by the same group employed an alternitive characterization for their patient cohort, categorizing into fast-progressing (FP-ALS) and slow-progressing ALS (SP-ALS), based on their ALSFRS-R rate of change [41,51]. In serum, they reported a decrease in hsa-miR-16–5p and an increase in hsa-miR-92a-3p correlating with higher rates of disability progression [41]. In a subsequent study using CSF, they found no differences in hsa-let-7c-5p, hsa-miR-9-3p, hsa-miR-196a-5p, hsa-miR-16–5p, hsa-miR-21–5p, or hsa-miR-92a-3p between SP-ALS and FP-ALS [51]. Additionally, Dobrowolny et al. also using this classification, found predictive value in higher levels of hsa-miR-206, hsa-miR-133a, and hsa-miR-151a-5p for SP-ALS vs FP-ALS [45].

Although we excluded longitudinal studies, Dobrowolny et al. met our eligibility criteria. Interestingly, they showed that in serum, early stages of ALS present high levels of hsa-miR-206 and hsa-miR-151a-5p, and lower levels of hsa-miR-133a, hsa-miR-199a-5p, and hsa-miR-423–3p [45]. Other studies presented follow-ups, including Andrade et al., who found no significant changes in hsa-miR-206 and hsa-miR-424 after 6 months (spinal-onset ALS, n = 19) and 12 months (spinal-onset ALS, n = 12) [28]. In serum, with a 3-month follow-up, Waller et al. found increased hsa-miR-143–3p, decreased hsa-miR-374–5p, and no differences in hsa-miR-206 in sALS (n = 22). Moreover, hsa-miR-143–3p was also significantly increased in lower-limb patients (n = 9) comparing other onset sites [32]. The true value of a biomarker resides not solely in its diagnostic potential, but also in its capacity to consistently and accurately reflect the progression of a disease and its response to a therapeutical treatment. Hence, we encourage that studies include, when possible, patient follow-ups, as well as the ALSFRS/-R as part of their cohort's characterization.

#### 4.1.7 Sample collection

Sample collection practices may be an important variability factor, as evidenced by reports showing the influence of miRNA expression depending on the time of day [69] and other conditions such as fasting. Only three studies explicitly noted collecting samples in the morning [21,24,30]. Another study, mentioned sampling was conducted during diagnosis [49], providing a specific time reference. Noteworthy, Waller et al. presented the most comprehensive data, with samples collected from their discovery cohort after an overnight fasting period, at the time of diagnosis, and prior to riluzole treatment initiation. In their validation cohort, sampling occurred regardless of fasting status, either at diagnosis or within three months thereafter, and encompassed individuals receiving riluzole treatment [32]. We recommend authors to document these pre-analytical variables related to the patient and to establish standardized sampling procedures and conditions for sample storage until processing. This approach will enable better control of potential sources of variability and increase reliability of results comparisons among researchers.

#### 4.1.8 Therapeutic interventions

Exploring the effects of riluzole, Waller et al. found no significant difference analyzing serum expression of hsa-miR-206, hsa-miR-143–3p, and hsa-miR-374b-5p between non-riluzole-treated (n = 13) and riluzole-treated (n = 10) patients [32]. Similarly, Vrabec et al., reported no differences in leukocyte expression of hsa-miR-143, hsa-miR-451, hsa-miR-338, hsa-miR-638, and hsa-let-7b between riluzole-treated (n = 31) and non-riluzole-treated (n = 37) subjects. They also observed (data not presented) a slight but statistically significant downregulation of hsa-miR-124a, hsa-miR-132, hsa-miR-206, and hsa-miR-663a, and upregulation of hsa-miR-9 in riluzole-treated patients [37]. In ALS management, patients receive multiple drugs, including psychotropics, anticholinergics, spasmolytics, benzodiazepines, opioids and non-opioids analgesics [70], among others. These medications, tipically undisclosed, may directly or indirectly influence miRNA expression individually or in combination. Only a few studies reported pharmacological intervention, focusing only on riluzole or edaravone. Furthermore, invasive procedures such as percutaneous endoscopic gastrostomy (PEG) and tracheostomy, which can induce an inflammatory state and physiological changes, are informed in only two studies [29,41]. Furthermore, lifestyle factors such as diet, exercise, and smoking are known to be miRNAome modulators [71]. Thus, documenting the patients' clinical history is crucial for unraveling these impacts and understanding disease-specific miRNAs amid therapeutic interventions and lifestyle choices.

### 4.2 Methodological variations

In this systematic review, we have focused on RT-qPCR-based studies, which are currently considered the gold standard for the quantification of steady-state mRNA levels due to their accuracy and sensitivity [72,73]. Yet, for this kind of analysis, it is essential to implement a suitable normalization strategy to correct experimental variations arising from inhibitory compounds, variations in reverse transcription efficiency, or disparities in the quality of the starting material [74]. Therefore, we included studies in our criteria that incorporated a method for such normalization, such as endogenously reference genes or spike-ins. Although our strategy has reduced the possibility of methodological variation, part of the reason for the lack of consistent patterns of gene expression could be attributed to the type of internal normalizer and other undetermined methodological variations [75].

Methodological variations are inherent to any technique, but it is important to note that there are recommendations for RT-qPCR technique and data reporting for publications [[76], [77], [78]] [[76], [77], [78]] [[76], [77], [78]] that authors should adhere to. Additionally, we recommend a few practices that future miRNA studies adopt, which may help standardize reporting and facilitate the generalizability of findings. First, we recommend that such studies report all results, significant and non-significant. Accurate accounting of all results is necessary to prevent systematic or publication bias in the literature, lending more confidence to the generalizability of findings in the field. Second, we recommend the reporting of anonymized, patient-level data. Such reporting allows for improved confidence in the statistical methods applied to synthesize evidence across multiple publications in future meta-analyses. Reporting of all underlying data also improves the reproducibility of analyses undertaken by the authors. Third, we recommend that all numerical data be reported as supplementary material in a machine-readable/algorithmically standardized format, such as a spreadsheet (e.g., CSV, Excel) or database (e.g., SQL, MongoDB), in addition to being plotted in figures and/or formatted tables in the manuscript. Since all the data necessary to implement our recommendations should already be readily available to the authors in writing their manuscripts and performing their analyses, our recommendations do not impose a significant burden on researchers while greatly enhancing the basic scientific principles of accessibility, rigor, and reproducibility.

### 4.3 Consistently dysregulated miRNAs and meta-analysis

Due to the risk of false positive results arising from the variations between studies, we focused our analysis on miRNAs with more than one dataset reporting the same dysregulated direction. The exclusion of inconsistently reported miRNAs and single datasets does not imply that they are irrelevant to the disease or unsuitable biomarkers; rather, their roles may be elucidated in future studies. This methodological approach resulted in 12 consistently dysregulated miRNAs, nine of which were represented either by multiple datasets from the same authors in the same publication: hsa-miR-3665, hsa-miR-4745–5p, hsa-miR-4530 [25], hsa-miR-34a-3p [49], or by the same authors in different publications: hsa-miR-146a-5p, hsa-miR-151a-5p, hsa-miR-10b-5p, hsa-miR-29b-3p, and hsa-miR-4454 [40,50].

Only hsa-miR-206 was consistently dysregulated across more than one tissue type and reported by several authors in independent works [23,28,29,32,37,46,48]. This pleiotropic molecule is specific to skeletal muscle and is associated with its development, cell differentiation, and regeneration, as well as various diseases, including ALS [[23], [79], [80], [81]]. The upregulation of hsa-miR-206 has been proposed as a regenerative mechanism triggered by muscle dystrophies, albeit insufficient for complete recovery [80,82]. Thus, as a potential biomarker, data points towards its role as a non-disease-causative molecule involved in muscle remodeling.

For the meta-analysis, we used highly conservative estimates of the variance in log_2_ fold changes of each miRNA when comparing ALS samples to control samples, as illustrated in the forest plots (Fig. 3, figs2.A-H). While each study reported results with p < 0.05, the statistical tests used to arrive at the p-values were highly heterogeneous, including parametric tests such as the Student t-test and nonparametric tests such as the Mann–Whitney *U* test. Due to the substantial heterogeneity in reporting of variances and confidence intervals, we used a sample size-based estimate of within-study variances [52] (Methods). Many of the resulting 95% confidence intervals shown in our forest plots overlap with 1 (no fold change). Despite our highly conservative estimates of variance, we were able to detect signals in our meta-analysis, contingent on the other assumptions made in our random effects models. As outlined in the Methods, we assumed that the log_2_ fold changes of each miRNA are drawn from a normal distribution. It is difficult to evaluate this assumption based on the small number of studies could analyze. Some of the miRNAs we examined did not reach statistical significance, as their 95% CIs overlap with 1. However, these analyses do not rule out the presence of a true difference between ALS and control groups and should be interpreted as requiring more data/publications to narrow the confidence intervals.

Our findings diverge from previous systematic reviews with meta-analysis centered on miRNAs from ALS [83,84], due to several methodological differences. First, we focus on liquid biopsy samples due to their accessibility and diagnostic value. Second, we undertake an in-depth epidemiological analysis and thoroughly discussing variations among the selected studies, offering strategies to standardize and enhance future studies. Third, we present our data at the individual data point level level. To our knowledge, this study represents the first systematic review to investigate the dysregulated expression of miRNAs derived from liquid biopsies from ALS patients compared with healthy controls by RT-qPCR.

### 4.4 Enriched pathways associated with the dysregulated miRNAs

From the 12 consistently dysregulated miRNAs, we retrieved pathways associated with ALS. The first enriched pathway, prion disease, exhibits a strong association with hsa-miR-338–3p. Evidence, both *in vivo* and *in vitro* suggests the involvement of prion-like mechanisms for the propagation, misfolding, and aggregation of proteins [85]. The following pathways, fatty acid biosynthesis, and metabolism were associated with the same miRNAs, hsa-miR-29b-3p, hsa-miR-10b-5p, hsa-miR-34a-3p and hsa-miR-338–3p. Alterations in lipid metabolism are known to participate in neurological disorders and have been directly linked to ALS [86]. ECM-receptor interaction pathway, associated with hsa-miR-29b-3p, and to lesser degrees, with hsa-miR-34a-3p, and hsa-miR-206. This pathway encompasses target genes involved in fibronectin, collagen, and laminin (data not shown). The dysregulation of ECM gene expression is common to neurodegenerative diseases as it provides structural and functional support and plays a role in the proliferation and differentiation of neuronal progenitors, as well as in axonal growth and guidance [87]. Another highly significant pathway was the adherens junction, associated with hsa-miR-183–5p, hsa-miR-151a-5p, and hsa-miR-29b-3p. Although adherens junctions have not been directly linked to ALS, they are implicated in other neurological diseases, as they are essential to the blood-brain barrier integrity [88]. Since we did not extract miRNA information on other neurological disease, we do not know the specificity of the our identified miRNAs to ALS. Consequently, these miRNAs and their associated pathways, could likely be shared with other neurological diseases.

## 5 Perspectives

Given the complexity of ALS, it is likely that a panel of biomarkers, encompassing non-coding RNAs, proteins, and metabolites, will be identified rather than a single molecular cue. This possibility has been explored by several groups, combining miRNAs with piwi-interacting RNA and transfer RNA [41,51], neurofilament light chain [89], and Nɛ-hexanoyl lysin (HEL) [43].

Importantly, since miRNA expression could be part of the pathomechanisms, it represents a potential therapeutic target. The overexpression of miRNAs could be treated using miRNA sponges, such as anti-miRNA oligonucleotides (AMOs), or small molecular inhibitors of specific miRNAs (SMIR). Conversely, deficiency of miRNAs could be supplemented by the administration of mimicking molecules [90]. However, the exploration of such therapeutic strategies is hindered by the lack of a reliable miRNA signature for ALS.

To address the challenges associated with stratification and low reproducibility, it is crucial to implement clear guidelines and standardized operating procedures. These guidelines should encompass the key variables discussed previously, including genetic characterization, comorbidity detection, clinical history, sampling and processing techniques, and screening methodologies. Furthermore, comprehensive data disclosure is essential. In addition to these measures, it is pertinent to establish protocolized assessments of disease progression and treatment response. To tackle the issue of limited sample sizes, well-coordinated international consortia with large cohorts and international biobanks adhering to good practices for the collection and storage of liquid biopsies can prove instrumental. Regionally, hospitals' involvement in collaborative initiatives to collect samples at the moment of diagnosis and in medical follow-ups could significantly aid research. Lastly, we are working on the creation of *ALSmiRBase*, a public database, such as EpimiRBase [91] for epilepsy, to promote transparency, sharing, and collaboration in the field. By implementing these suggestions, we can enhance the robustness and translatability of biomarker research for ALS.

## 6 Conclusion

Our study provides valuable insights into research aimed at identifying miRNAs as biomarkers for ALS. We have compiled the most comprehensive list to date of the results of every miRNA screened by PCR in liquid biopsy samples compared with healthy controls. This includes miRNAs screened only once, those with conflicting expression across studies, and those consistently showing the same direction of dysregulation. Furthermore, we identified and discussed the substantial methodological variations that contribute to inconsistencies between studies. Our meta-analysis revealed a low likelihood of publication bias or p-value manipulation, indicating the reliability and integrity of the analyzed studies. Our work offers insights into the current status of the field and enhances the understanding of the challenges and opportunities in identifying reliable miRNA biomarkers for ALS. We anticipate that our study will inspire protocol standardization and facilitate future research in the quest for dependable miRNA biomarkers for ALS.

## Data and code availability

All data and code to reproduce our meta-analysis are included in a Zenodo repository (10.5281/zenodo.8139857) as well as in the Supplemental Materials.

## Declaration of interest

None.

## Funding

The authors did not receive direct funding for this research. However, we would like to thank the Ministry of Health of Brazil (325000.475256/2017-54) and INOVA FIOCRUZ for their support. NVD also acknowledges the support from the Passan Foundation.

## CRediT authorship contribution statement

**Hemerson Casado Gama:** Writing – original draft, Visualization, Validation, Investigation, Formal analysis, Data curation, Conceptualization. **Mariana A. Amorós:** Writing – review & editing, Writing – original draft, Visualization, Validation, Resources, Methodology, Investigation, Formal analysis, Data curation, Conceptualization. **Mykaella Andrade de Araújo:** Writing – review & editing, Writing – original draft, Validation, Resources, Investigation, Formal analysis, Data curation, Conceptualization. **Congzhou M. Sha:** Writing – review & editing, Writing – original draft, Visualization, Validation, Software, Resources, Methodology, Investigation, Formal analysis, Data curation, Conceptualization. **Mirella P. S. Vieira:** Investigation. **Rayssa G.D. Torres:** Investigation. **Gabriela F. Souza:** Investigation. **Janaína A.Junkes:** Investigation. **Nikolay V. Dokholyan:** Writing – review & editing, Visualization, Supervision, Conceptualization. **Daniel Leite Góes Gitaí:** Writing – review & editing, Visualization, Supervision, Methodology, Conceptualization. **Marcelo Duzzioni:** Writing – review & editing, Visualization, Supervision, Conceptualization.

## Figures and Tables

**figs1:**
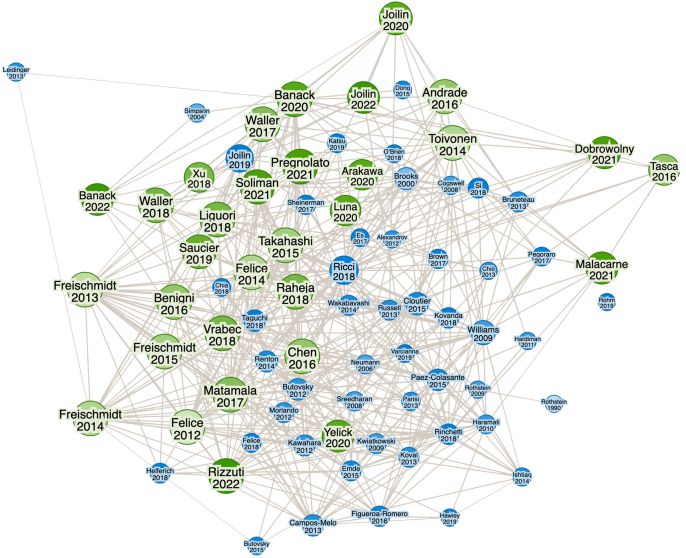


**figs2:**
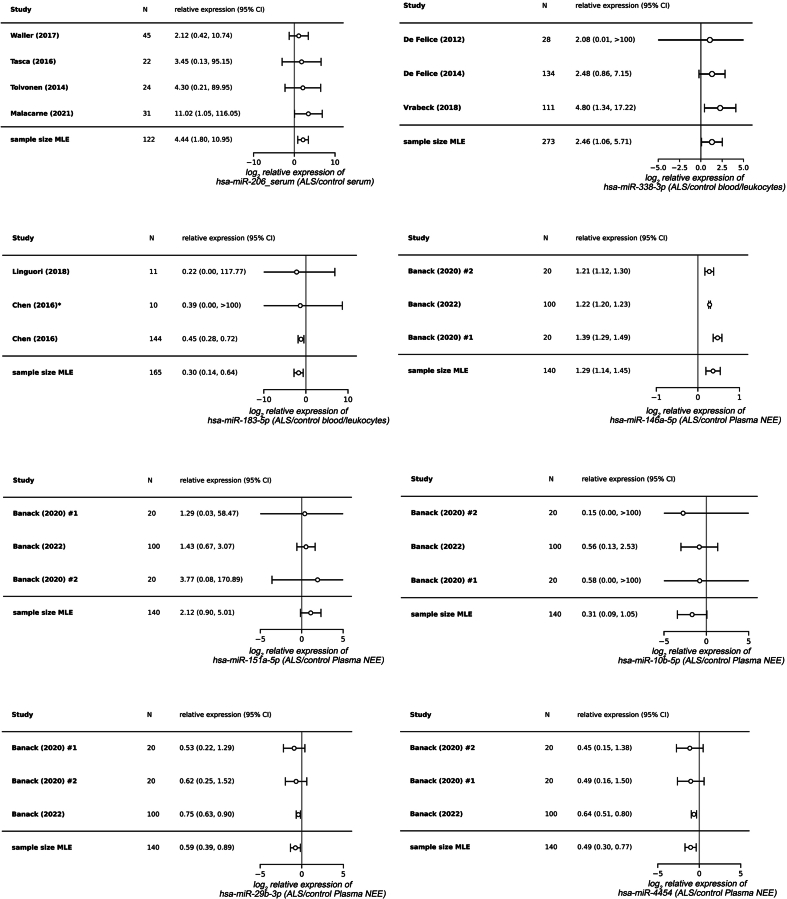


## Appendix A Supplementary data

**ADDITIONAL FILE 1:**Fig. S1**:** Supplemental search performed via ResearchRabbit (February 2023) retrieved a total of 2783 related publications and identified no additional articles. Represented in green are our 31 selected studies, and in blue are the most relevant publications associated with them.

**ADDITIONAL FILE 2:**Table S1**:** Extracted information from the 31 articles comparing miRNAs expression between ALS patients and healthy controls via PCR. Abbreviations: ALS-FP, fast-progressing ALS; ALS-SP, slow-progressing ALS; ALSFRS/-R, Amyotrophic Lateral Sclerosis Functional Rating Scale/-Revised; ALS-PDC Kii Med Manual, medical manual for ALS and Parkinsonism-dementia complex of the Kii Peninsula of Japan; CSF, cerebrospinal fluid; El-Escorial/-R, -Revised; ENMG, electroneuromyography; EVs, extracellular vesicles; HC, healthy control; K-ALS, Kii peninsula ALS; K-residents, Kii peninsula control residents. Symbols: ♦, Calculated based on the article's data; ♦♦, Age- and sex-matched; #, Information of patient cohort from 10.1093/brain/awu249; ##, Information from correspondence with authors.

**ADDITIONAL FILE 3:**Table S2**:** Extracted information on the expression of every miRNA analyzed via PCR and compared to healthy control. Abbreviations: ALS-FP, fast-progressing ALS; ALS-SP, slow-progressing ALS; CSF, cerebrospinal fluid; EVs, extracellular vesicles; HC, healthy control; NEE, neural-enriched EVs. Symbols: ↑, upregulated; ↓, downregulated; =, unaltered; #, Authors kindly provided information on the miRNA’ -3p or -5p; ##, These miRNAs have identical sequences, making it impossible to distinguish them from each other, as a result, they are considered as the same miRNA and counted only once.

**ADDITIONAL FILE 4:**Table S3**:** Analysis of miRNAs stratified by tissue source. Consistent miRNAs are molecules with the same expression reported in more than one dataset. Inconsistent miRNAs are molecules with different expressions reported in more than one dataset. Abbreviations: ALS-FP, fast-progressing ALS; ALS-SP, slow-progressing ALS; CSF, cerebrospinal fluid; EVs, extracellular vesicles; HC, healthy control; NEE, neural-enriched EVs. Symbols: ↑, upregulated; ↓, downregulated; =, unaltered; #, Authors kindly provided information on the miRNA’ -3p or -5p; ##, These miRNAs have identical sequences, making it impossible to distinguish them from each other, as a result, they are considered as the same miRNA and counted only once.

**ADDITIONAL FILE 5**Fig. S2**:** The forest plots for **(A)** hsa-miR-206 (serum); **(B)** hsa-miR-338–3p (peripheral blood/leukocytes); **(C)** hsa-miR-183–5p (peripheral blood/leukocytes); **(D)** hsa-miR-146a-5p (plasma NEE); **(E)** hsa-miR-151a-5p (plasma NEE); **(F)** hsa-miR-10b-5p (plasma NEE); **(G)** hsa-miR-29b-3p (plasma NEE); **(H)** hsa-miR-4454 (plasma NEE). *The initial pilot study performed by the authors in this paper. #1 and #2 are two independent cohorts reported in the same manuscript.
